# FMRI study of implicit emotional face processing in patients with MDD with melancholic subtype

**DOI:** 10.3389/fnhum.2023.1029789

**Published:** 2023-02-27

**Authors:** Almira Kustubayeva, James Eliassen, Gerald Matthews, Erik Nelson

**Affiliations:** ^1^Department of Psychiatry and Behavioral Neuroscience, University of Cincinnati, College of Medicine, Cincinnati, OH, United States; ^2^Center for Cognitive Neuroscience, Department of Biophysics, Biomedicine, and Neuroscience, Al-Farabi Kazakh National University, Almaty, Kazakhstan; ^3^National Centre for Neurosurgery, Astana, Kazakhstan; ^4^Robert Bosch Automotive Steering, Florence, KY, United States; ^5^Department of Psychology, George Mason University, Fairfax, VA, United States

**Keywords:** depression, brain activity, emotional face processing, functional MRI, melancholic subtype

## Abstract

**Introduction:**

The accurate perception of facial expressions plays a vital role in daily life, allowing us to select appropriate responses in social situations. Understanding the neuronal basis of altered emotional face processing in patients with major depressive disorder (MDD) may lead to the appropriate choice of individual interventions to help patients maintain social functioning during depressive episodes. Inconsistencies in neuroimaging studies of emotional face processing are caused by heterogeneity in neurovegetative symptoms of depressive subtypes. The aim of this study was to investigate brain activation differences during implicit perception of faces with negative and positive emotions between healthy participants and patients with melancholic subtype of MDD. The neurobiological correlates of sex differences of MDD patients were also examined.

**Methods:**

Thirty patients diagnosed with MDD and 21 healthy volunteers were studied using fMRI while performing an emotional face perception task.

**Results:**

Comparing general face activation irrespective of emotional content, the intensity of BOLD signal was significantly decreased in the left thalamus, right supramarginal gyrus, right and left superior frontal gyrus, right middle frontal gyrus, and left fusiform gyrus in patients with melancholic depression compared to healthy participants. We observed only limited mood-congruence in response to faces of differing emotional valence. Brain activation in the middle temporal gyrus was significantly increased in response to fearful faces in comparison to happy faces in MDD patients. Elevated activation was observed in the right cingulate for happy and fearful faces, in precuneus for happy faces, and left posterior cingulate cortex for all faces in depressed women compared to men. The Inventory for Depressive Symptomatology (IDS) score was inversely correlated with activation in the left subgenual gyrus/left rectal gyrus for sad, neutral, and fearful faces in women in the MDD group. Patients with melancholic features performed similarly to controls during implicit emotional processing but showed reduced activation.

**Discussion and conclusion:**

This finding suggests that melancholic patients compensate for reduced brain activation when interpreting emotional content in order to perform similarly to controls. Overall, frontal hypoactivation in response to implicit emotional stimuli appeared to be the most robust feature of melancholic depression.

## Introduction

Major depressive disorder (MDD) is a multidimensional disorder characterized by altered emotional experience and processing. It is one of the more prevalent psychiatric disorders and imposes major personal and social burdens (Kessler et al., 2005). Indeed, depression is the leading cause of disability worldwide (World Health Organization, 2017). The adverse impacts of the disorder are experienced disproportionately by women, for whom the prevalence of depression is approximately twice as high than in men (Salk et al., 2017). Multiple brain systems and processes are implicated in major depression including abnormalities in frontal, limbic parietal, and default brain networks (Kaiser et al., 2015; Drysdale et al., 2017).

Understanding these abnormalities in brain functioning may contribute to developing effective therapy for depression (Keedwell et al., 2010; Roseman et al., 2018; Stroud et al., 2018; Drobisz and Damborská, 2019; Gorka et al., 2019; Fisher et al., 2022; Pizzagalli and Roberts, 2022).

### Depression and emotional stimulus processing

Brain mechanisms may underpin clinically significant abnormalities in processing emotional stimuli. Patients with MDD exhibit deficits in emotional face processing and emotion recognition (Bouhuys et al., 1996; Bourke et al., 2010; Brockmeyer et al., 2013). They also show “mood-congruent” biases in attention and cognition. That is, depressed individuals tend to attend to negative rather than positive stimuli, interpret ambiguous stimuli negatively, and selectively recall negative memories (Leppänen, 2006; Gollan et al., 2008; Kustubayeva et al., 2012; LeMoult and Gotlib, 2019). These biases may disrupt social functioning and contribute to the onset and maintenance of depression (Gray et al., 2021).

There have been numerous neuroimaging studies of brain response to emotive stimuli in patients with MDD (Davidson et al., 2002; Leppänen, 2006; Wagner et al., 2006; Groenewold et al., 2013; Li and Wang, 2021; Kustubayeva et al., 2022). Such studies commonly manipulate images of facial emotion (Bourke et al., 2010), although verbal and pictorial stimuli are also utilized. Results are complex but some general trends toward differences between patients and healthy controls have emerged. These include increased activation in limbic regions (Mayberg, 1997, 2003; Anand et al., 2005; Leppänen, 2006; Zhong et al., 2011), as well as enhanced amygdala response to both positively and negatively valenced emotional stimuli (Young et al., 2018). Depressed individuals also tend to show stronger response in areas associated with emotional appraisal and salience processing including insula, anterior cingulate cortex (ACC) and other paralimbic areas (Jaworska et al., 2015; Burger et al., 2017; Li and Wang, 2021; Pizzagalli and Roberts, 2022). In addition to abnormalities in early stages of emotive stimulus processing, depression is linked to brain regions supporting later emotion-regulation processes, such as dorsal left prefrontal cortex (DLPFC; Groenewold et al., 2013). Behavioral data show that emotion-regulation is often deficient in depression (Bylsma et al., 2008; Liu and Thompson, 2017). Impairment in emotion-regulation is also suggested by effects of depression on the functional connectivity of the amygdala and areas of prefrontal cortex (Pizzagalli and Roberts, 2022).

Some functional magnetic resonance imaging (fMRI) studies show depression effects that generalize across positive and negative stimuli (Li and Wang, 2021). However, numerous studies support the “mood-congruent hypothesis” which posits that depressed patients exhibit increased responses to negative emotions and decreased responses to positive emotions (Leppänen et al., 2004; Leppänen, 2006; Suslow et al., 2010; Stuhrmann et al., 2011; Zhong et al., 2011). Meta-analyses have confirmed that MDD is linked most strongly to greater ACC activation in response to negative emotional stimuli (Groenewold et al., 2013, Li and Wang, 2021). However, these two meta-analyses found conflicting outcomes for ACC response to positive stimuli. Li and Wang (2021) suggest that depressed individuals may be attentive to positive stimuli as an adaptive strategy that aims to alleviate persistent depressive emotion. Prefrontal cortex activation may regulate processing of facial expression in emotion (Van Wingen et al., 2011; Manelis et al., 2019). Groenewold et al. (2013) meta-analysis also found that MDD is associated with decreased DLPFC response to negative stimuli and increased orbitofrontal (OFC) response to positive stimuli, suggesting impairments in voluntary emotion-regulation.

### Clinical population heterogeneity and depression effects

The reviews cited (Leppänen, 2006; Groenewold et al., 2013; Li and Wang, 2021; Pizzagalli and Roberts, 2022) acknowledge the inconsistency of results across studies. In part, inconsistency may reflect associations between depression and multiple, overlapping emotion-processing brain networks (Groenewold et al., 2013; Li and Wang, 2021). Different task demands such as the need for executive control, affect which networks are activated, and consequently the pattern of differences in brain response between depressed individuals and healthy controls (Kustubayeva et al., 2020; Pizzagalli and Roberts, 2022). Methodological differences between studies may also lead to variation in outcomes (Jaworska et al., 2015). Another relevant factor is heterogeneity in clinical populations; outcomes may be different according to factors such as symptom severity, depression subtype, and demographic characteristics (Lawrence et al., 2003; Jaworska et al., 2015; Müller et al., 2017; Gong et al., 2020). In this study, we considered two such factors: subtype and gender.

Diagnostic And Statistical Manual of Mental Disorders, Fifth Edition (DSM-5) includes several subtypes for a major depressive episode (MDE), including MDE with melancholic features, MDE with atypical features, MDE with anxious distress, and MDE with psychotic features (American Psychiatric Association, 2013, DSM-5). The melancholic subtype is characterized by blunted affective reactivity to emotional stimuli and significant anhedonia and a specific presentation of neurovegetative symptoms. Another phenomenological subtype, atypical features, consists of symptoms that could be considered opposite to the melancholic subtype such as increased sleep, higher appetite/weight gain, and mood reactivity. In this study, we chose to focus solely on the melancholic subtype to reduce the amount of heterogeneity that may be present in the findings particularly if melancholic and the other subtype patients were included in a single group of depressed patients. Behavioral studies reported profound cognitive dysfunction in patients with melancholic features compared to patients without melancholic features and healthy participants (Bosaipo et al., 2017; Esposito and Buoli, 2020). Behavioral differences in depression heterogeneity are reflected in underlying neurocognitive processes. For instance, multimodal neuroimaging distinguishes brain activation dysfunction with blunted feedback negativity amplitude and diminished activation in ventral striatum for melancholic phenotype of MDD (Fountoulakis et al., 2004; Foti et al., 2014). Because of the heterogeneity of depressive subtypes this study recruited a homogenous population of melancholic patients.

The greater susceptibility of women to MDD (Kuehner, 2003, 2016; Salk et al., 2017) may relate in part to differences between men and women in the brain circuitry that underlies emotional processing. Neuroimaging studies in healthy participants report anatomical (Good et al., 2001; Gur et al., 2002a) and functional (Wager et al., 2003; Stevens and Hamann, 2012; Weisenbach et al., 2014) differences between men and women in brain regions involved in emotion. Meta-analyses suggest that males and females may differ in both limbic and cortical activations during emotional processing (Wager et al., 2003; Fusar-Poli et al., 2009). Additionally, men display greater hemisphere lateralization in emotion processing (Bourne and Jonauskaite, 2015). Behavioral studies find that depressed women are more sensitive to negative emotion compared to depressed men (Mufson and Nowicki, 1991; Thayer and Johnsen, 2000; Gotlib et al., 2004; Bourke et al., 2010). Women also differ from men in their strategies for emotion-regulation, expressed in both subjective and neuropsychological outcome measures (Domes et al., 2010; Kustubayeva et al., 2013; Tolegenova et al., 2014). These factors may contribute to a greater persistence of depressive symptoms in women (Bouhuys et al., 1996). However, although many studies report sex differences in emotional network activity during emotional face processing in healthy participants, there are few studies of the relationship of these phenomena to increased susceptibility of women to MDD (Wright et al., 2009; Briceño et al., 2015; Jenkins et al., 2018). It is important, therefore, to investigate the neurobiological basis for depressive disorders in women, controlling for MDD phenomenology because sex differences may be subtype-specific.

### Aims of study

The present study is the first to investigate the difference**s** in brain activation during emotional face perception in patients with the melancholic subtype of MDD in comparison to healthy participants and to examine sex differences in a homogenous MDD group. We *aimed* to distinguish the pattern of brain activation to positive and negative emotions during a face processing task between patients meeting the criteria for the melancholic subtype of MDD and healthy participants. Additionally, we investigated how the neural correlates of depression are influenced by sex. We used a modified version of an established emotional faces task (Gur et al., 1992) that requires implicit emotion processing. We expected that the implicit emotional face task would allow us to investigate altered neural responses to unconsciously processed emotions specifically in MDD patients with melancholic features. Our hypotheses were: (1) decreased prefrontal cortical activation and increased limbic activation in patients with melancholic depression compared to healthy participants; (2) increased neural response to negative relative to positive emotional faces in participants with MDD consistent with the mood-congruent hypothesis; (3) more pronounced frontal hypoactivation and mood-congruence in depressed women; (4) correlations between severity of symptoms and BOLD responses associated with depression. We used a modified version of an established emotional faces task (Gur et al., 1992) with implicit emotion processing. We expected that the implicit emotional face task would allow us to investigate the altered reaction to unconsciously perceived emotions specifically in MDD patients with melancholic features.

## Materials and methods

### Participants

Thirty participants who met DSM-IV criteria for a current major depressive episode with melancholic features (MDD, 15 female, average age = 43.67, SD = 8.59) and 21 healthy participants (HP, 12 female, average age = 37.65, SD = 11.83) were recruited for the study via advertising, physician referral, and word of mouth. Data from four men in the HP group were excluded due to uncorrectable fMRI motion artifacts. All participants provided written informed consent prior to participating in the study. The study was approved by the Institutional Review Board (IRB) of the University of Cincinnati. Demographic, clinical and behavioral characteristics are summarized in Table 1. Depressive symptoms were assessed by using the clinician rated version of the Inventory for Depressive Symptoms (IDS-CR, Rush et al., 1986). All patients were diagnosed as meeting DSM-IV criteria for MDD with melancholic features using the Structured Clinical Interview for the DSM-IV (SCID) conducted by the study psychiatrist (American Psychiatric Association, 2013, Diagnostic and Statistical Manual of Mental Disorders, DSM-IV, First et al., 1996). Additionally, a measure of the level of melancholic symptoms was determined for each MDD participant using the sum of items on the IDS that pertain to DSM-IV criteria for melancholic features specifier (items 3, 8, 9, 10, 11, 13, 21, 23, 31).

**TABLE 1:** Demographic and clinical data.

	MDD			HC		
		**F**	**M**		**f**	**M**
N	30	15	15	17	12	5
Age	43.52 SD = 8.49	44.64 SD = 9.93	42.43 SD = 8.45	37.65 SD = 11.83	37.83 SD = 12.78	37.2 SD = 10.5
**Ethnicity**						
African American	25%			29.4%		
Caucasian	64.29%			58.8%		
Asian	3.57%			5.9%		
Hispanic	3.57%			5.9%		
Native American	3.57%					
Inventory for Depressive Symptomatology	37.32 SD = 7.49	37.46 SD = 7.29	37.26 SD = 7.24			
Melancholic symptom score		12.67 SD = 2.77	13.07 SD = 4.40			

Participants were included if they had taken no antidepressant medications or supplements for 2 weeks prior to screening (4 weeks for fluoxetine). They were required to abstain from all psychotropic medications for two days prior to the scanning dates. Healthy participants were included if they had no personal or first-degree family history of DSM-IV Axis I psychiatric disorders (including substance use disorders), no current unstable medical conditions, and no lifetime history of neurological disorders. To confirm the absence of past or present psychiatric disorders, healthy participants were screened using the SCID for DSM-IV by the same study psychiatrist.

### Stimuli and task design

**FIGURE 1:**
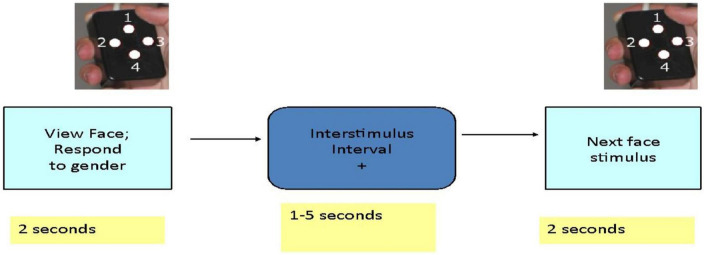
Schematic view of emotional faces task.

Each participant was given oral and written instructions and allowed to briefly practice the task with face images that were not used during scanning. The behavioral task was programmed in E-Prime.^1^ Subjects participated in a 14-min scanning session during which they viewed 120 different facial identities each depicting a particular emotion (sadness, happiness, or fear) or a neutral expression (Penn Emotional Faces, Gur et al., 1992). Subjects were asked to decide the gender rather than the emotional expression of each face and were asked to press one of two buttons with their right thumb to indicate their answer. Subjects viewed 30 faces of each emotion or neutral expression presented in random order. Facial stimuli were presented for 2 s each. The interval between stimuli varied between 3 and 8 s with an average interval of 4.9 s and participants viewed a fixation cross during this interval (Figure 1).

### Image acquisition

Participants were scanned using a 4.0 Tesla Varian Unity INOVA Whole Body MRI/MRS system (Varian Inc., Palo Alto, CA, USA) at the Center for Imaging Research at the University of Cincinnati College of Medicine. To provide anatomical localization for activation maps, a high-resolution T1-weighted anatomical image was acquired using modified driven equilibrium Fourier transform, MDEFT (T_*MD*_ = 1.1 s, TR = 13 ms, TE = 6 ms, FOV = 256 mm × 192 mm × 192 mm, matrix 256 × 192 × 96 mm, voxel resolution 1 mm × 1 mm × 2 mm, flip angle = 20°), zero-filled to 1 mm × 1 mm × 1 mm during reconstruction. For fMRI measurements, we acquired T2*-weighted gradient-echoplanar images (EPI) (TR/TE = 3,000/29 ms, FOV = 208 mm × 208 mm, matrix 64 × 64, slice-thickness 5 mm, flip angle = 75°, resolution 3.25 mm × 3.25 mm × 5 mm) during presentation of faces.

### Preprocessing of fMRI data and statistical analysis

Following acquisition, images were reconstructed using software developed in IDL (Interactive Data Language), which converts raw FID files into AFNI format (Analysis of Functional NeuroImages; Cox, 1996, Cox and Hyde, 1997). In AFNI, MDEFT (structural), and EPI (functional) images were co-registered using scanner coordinates. Functional images were corrected for motion using a six-parameter rigid body transformation with the 3dvolreg routine. After realignment, each dataset was reviewed for uncorrected motion and individual images were censored from further processing if uncorrectable image movement was detected. The functional data were then transformed to Talairach space using the participant’s anatomical image and the @auto_tlrc routine. Binary masking was applied to each image to remove pixels outside the brain. Activation maps were created using a deconvolution algorithm that compared the actual hemodynamic response to a canonical response function (e.g., gamma function). AFNI then generated an estimate of the ‘fit coefficient’ (i.e., beta weight or scaling factor) describing the magnitude of the hemodynamic response relative to the average signal intensity. Low-frequency components of the signal, including linear and quadratic drift, were also removed. In total, image processing included co-registration, motion correction, 6 mm blur, normalization to Talairach space, resampling (3 mm^3^), and event-related analysis. Statistical analyses were performed using multivariate modeling (3dMVM, Chen et al., 2014). In order to define significant activation, we thresholded the activation maps at an uncorrected *p*-value less than 0.005 and a cluster of 37 nearest neighbor voxels. This yielded a cluster-corrected statistical threshold of *p* ≤ 0.005. Additionally, average BOLD signal activity from Regions of Interest (ROIs) were extracted and analyzed by using IBM Corp (2020).

### Statistical analysis of behavioral data

Two analyses of the behavioral data were conducted using one-way ANOVA with IBM Corp (2020). In the first analysis reaction time was the dependent variable, and group, sex, and emotion were the independent factors. In the second analysis the number of errors was the dependent variable with group, sex, and emotion as the independent factors.

## Results

### Demographics and behavioral data

Analyses of demographic variables showed no significant differences between depressed subjects and healthy participants (Table 1). Severity of depression as measured by IDS score was similar in female and male MDD groups.

**FIGURE 2:**
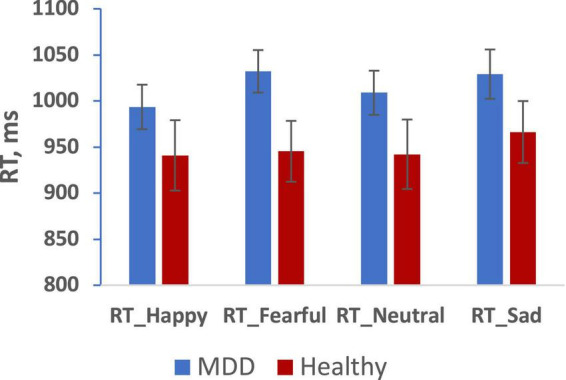
Mean reaction time for happy, fearful, neutral, sad faces in MDD (blue), and Healthy (red) groups. (Mean ± SE). *p < 0.05.

**FIGURE 3:**
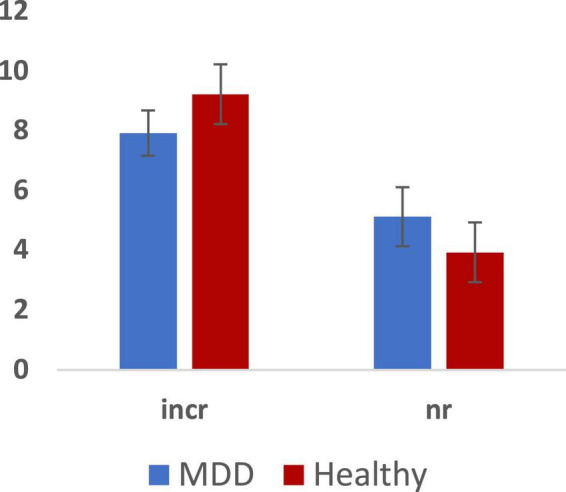
Total errors across MDD and Healthy groups (Mean ± SE): incorrect responses (incr) and non-response (nr).

Results with reaction time (RT) to emotional faces for correct responses presented on Figures 2, 3.

We analyzed RT and number of errors using repeated measures ANOVA with group, sex, and emotion as independent predictors. We observed a main effect of emotion (*F* = 3.132, *p* = 0.028, 0.176) but no main effects of group or sex on RT. Furthermore, we observed no 2-way or 3-way interactions among group, sex, and emotion. Paired sample tests to clarify the effect of emotion showed significantly slower RT for both fearful and sad faces compared to happy faces (*p* < 0.05). There were no significant effects in the analysis of the number of errors.

### Functional imaging data

Comparison of BOLD signal intensity between healthy participants and MDD patients with melancholic depression

**FIGURE 4:**
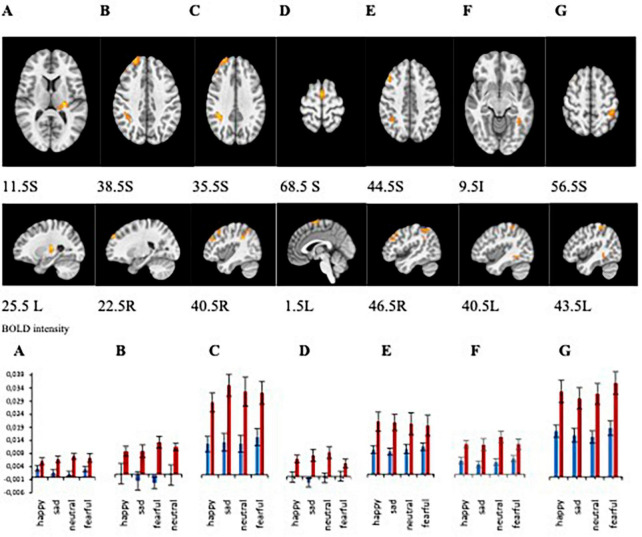
**(A–G)** Brain regions that showed significant group differences between MDD and Healthy for presentations of happy, sad, fearful, and neutral faces [**(A)** left thalamus/left claustrum, **(B)** right superior frontal gyrus, **(C)** right supramarginal gyrus, **(D)** left superior frontal gyrus, **(E)** right middle frontal gyrus, **(F)** right fusiform gyrus, **(G)** left inferior parietal lobule]. Plots for ROI that displayed a significant difference between BOLD intensity in MDD (blue) and HP (red) groups in the corresponding brain areas (*p* < 0.005).

Statistical analyses of the BOLD signal were conducted using 3dMVM with 2 between-subject variables (group: “MDD” and “HP,” sex: “female” and “male”) and 1 within-subject variable (emotion: “happy,” “neutral,” “fearful,” “sad”). The ANOVAs revealed a significant group effect in the left thalamus, right supramarginal gyrus, right and left superior frontal gyrus, right middle frontal gyrus, and left fusiform gyrus (*p* ≤ 0.005) (Figure 4A and Table 2). BOLD signal intensity was higher in healthy participants compared to the MDD group in the regions mentioned above. The directions of the effects are shown in Figures 4A–G of the average BOLD intensity in ROIs extracted from clusters that showed these significant effects.

**TABLE 2:** Regions with significant group effect.

Cluster #	Cluster size (# voxels)	Center of mass			Peak activation	Brodmann area
		**X**	**Y**	**Z**		
		**(Local peak activation)**				
1	123	+25.5	+22.5	+11.5	Left thalamus/left claustrum, left insula	
2	109	−22.5	−55.5	+38.5	Right superior frontal gyrus	BA 9,8
3	109	−40.5	+40.5	35.5	Right supramarginal gyrus	BA 40
4	66	+1.5	+4.5	+68.5	Left superior frontal gyrus	BA 6
5	41	−46.5	−22.5	+44.5	Right middle frontal gyrus	BA 8
6	38	+40.5	+49.5	−9.5	Left fusiform gyrus	BA 37
7	37	+43.5	+40.5	+56.5	Left inferior parietal lobule	BA 40

ROI analyses from those clusters showed significantly decreased BOLD intensity in the MDD group compared to the healthy group for each emotional valence in the left and right superior frontal gyrus (left: *F* = 11.243, *p* = 0.002 for happy; *F* = 16.166, *p* < 0.001 for sad; *F* = 11.769, *p* < 0.001 for neutral; *F* = 10.243, *p* = 0.003 for fearful; right: *F* = 10.841, *p* = 0.002 for happy, *F* = 7.386, *p* = 0.009 for sad, *F* = 20.342, *p* < 0.001 for neutral, and *F* = 8.959, *p* = 0.004 for fearful); in the left thalamus for sad (*F* = 9.840, *p* = 0.003), fearful (*F* = 4.856, *p* = 0.033), and neutral (*F* = 23.395, *p* < 0.001) faces; in the left fusiform gyrus for happy (*F* = 10.076, *p* = 0.003), sad (*F* = 15.186, *p* < 0.001), neutral (*F* = 6.500, *p* = 0.014), and fearful (*F* = 5.216, *p* = 0.027) faces; in the right supramarginal gyrus for happy (*F* = 10.841, *p* = 0.002), sad (*F* = 10.680, *p* = 0.002), neutral (*F* = 4.238, *p* < 0.001), and fearful (*F* = 8.959, *p* = 0.004) faces; in right middle frontal gyrus for happy (*F* = 8.482, *p* = 0.006), sad (*F* = 14.589, *p* < 0.001), neutral (*F* = 12.087, *p* < 0.001), and fearful (*F* = 4.948, *p* = 0.021) faces; and left inferior parietal lobule happy (*F* = 11.408, *p* = 0.002), sad (*F* = 9.360, *p* = 0.004), neutral (*F* = 15.633, *p* < 0.001), and fearful (*F* = 13.642, *p* = 0.001) faces (Figure 4B). Our results showed decreased activation in the frontal areas in response to all faces in patients with the melancholic subtype of MDD in comparison to healthy participants. Thus, activation was not dependent on emotional valence suggesting a lack of emotional bias. The main effect of sex showed significant activation in the left middle frontal gyrus and right precuneus where women exhibited greater activation than men.

#### Emotion effects on BOLD response

**FIGURE 5:**
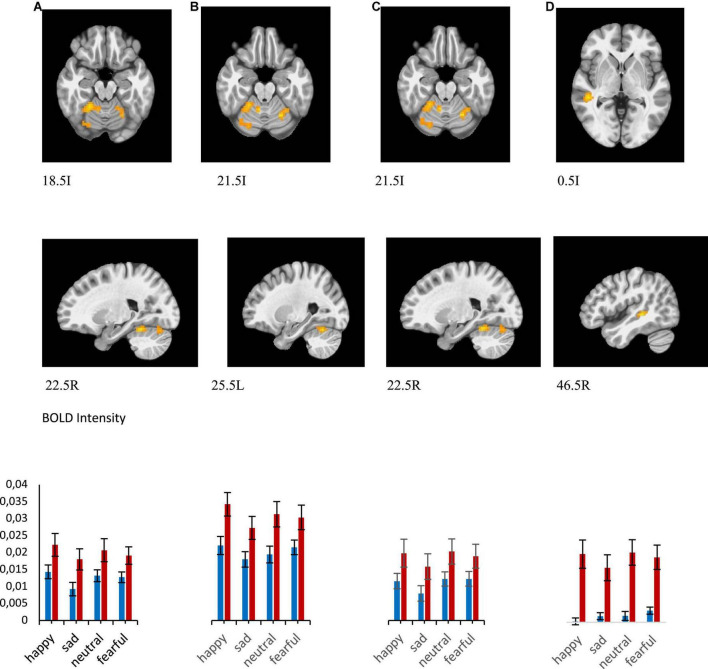
Brain regions that showed significant emotion effect in all participants for presentations of happy, sad, fearful, and neutral faces [**(A)** right culmen, happy > sad, Healthy > MDD, **(B)** left declive, happy > sad, Healthy > MDD, **(C)** right declive, **(D)** right middle temporal gyrus]. BOLD intensity during happy, sad, neutral, and fearful faces perception in MDD and Healthy groups in A and B brain areas (*p* < 0.005).

There was a significant main effect of emotion (*F* = 15.696, *p* = 0.000, 0.254) in right culmen, left declive, right declive, and right middle temporal gyrus (Figure 5 and Table 3). Pairwise *post-hoc* comparisons of the right culmen activation levels indicated that for MDD patients activation in response to happy or fearful faces was greater than to sad faces (happy > sad, *p* = 0.018, fearful > sad, *p* = 0.025). For the healthy group there were no significant pairwise differences. For the right middle temporal gyrus activation level was higher to fearful and sad faces in comparison to happy face (fearful > happy, *p* < 0.001, sad > happy, *p* = 0.024 for all participants, fearful > happy, *p* = 0.002 for MDD group). Our analysis of the group by emotion interaction did not identify significant regions of activation.

**TABLE 3:** Regions with significant emotion effect.

Cluster #	Cluster size (# voxels)	Center of mass			Peak activation	Brodmann area
		**X**	**Y**	**Z**		
		**(Local peak activation)**				
1	72	−25.5	+49.5	−18.5	Right culmen	
2	53	+25.5	+61.5	−21.5	Left declive	
3	39	−22.5	+76.5	−21.5	Right declive	
4	38	−46.5	+34.5	−0.5	Right middle temporal gyrus	

#### Sex differences in BOLD response in MDD group

**FIGURE 6:**
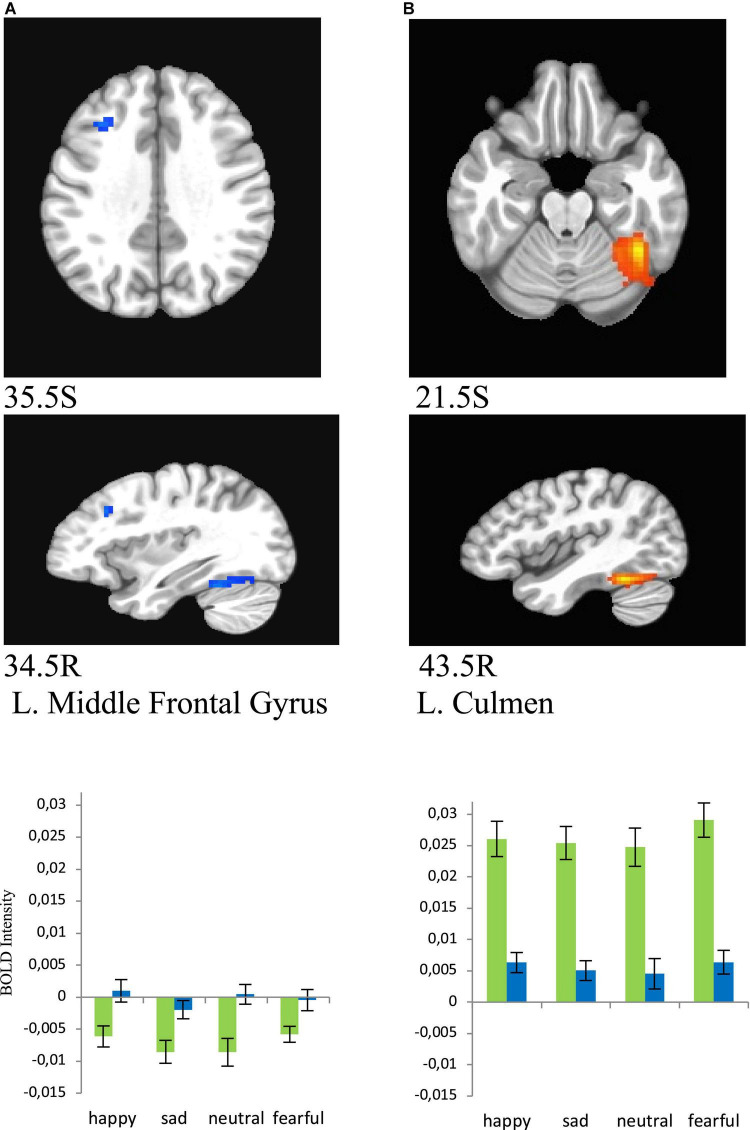
Brain regions that showed significant sex effect in MDD participants for presentations of happy, sad, fearful, and neutral faces [females – green color, males – blue color; **(A)** left middle frontal gyrus/BA8 men > women, **(B)** left culmen women > men]. BOLD intensity during happy, sad, neutral, and fearful faces perception in MDD groups for females and males in panels **(A,B)** brain areas (*p* < 0.005).

Statistical results of the comparison of BOLD intensity between female and male patients with MDD are shown in Figure 6 and Table 4.

**TABLE 4:** Regions with significant sex effect in MDD group.

Cluster #	Cluster size (# voxels)	Center of mass			Peak activation	Brodmann area
		**X**	**Y**	**Z**		
		**(Local peak activation)**				
1	193	+43.5	+49.5	−21.5	Left culmen	
2	49	+34.5	−25.5	+35.5	Left middle frontal gyrus	BA 9

Women showed significantly lower activation than men in the left middle frontal gyrus (BA8) (happy (*F* = 9.354, *p* = 0.005), sad (*F* = 8.835, *p* = 0.006), neutral (*F* = 12.402, *p* = 0.001), and fearful (*F* = 7.103, *p* = 0.013), and significantly greater activation in left culmen for all conditions [happy (*F* = 39.626, *p* = 0.000), sad (*F* = 46.976, *p* = 0.000), neutral (*F* = 28.775, *p* = 0.000), and fearful (*F* = 49.716, *p* = 0.000), Figure 6].

#### Interactive effects of sex and group on BOLD response

**FIGURE 7:**
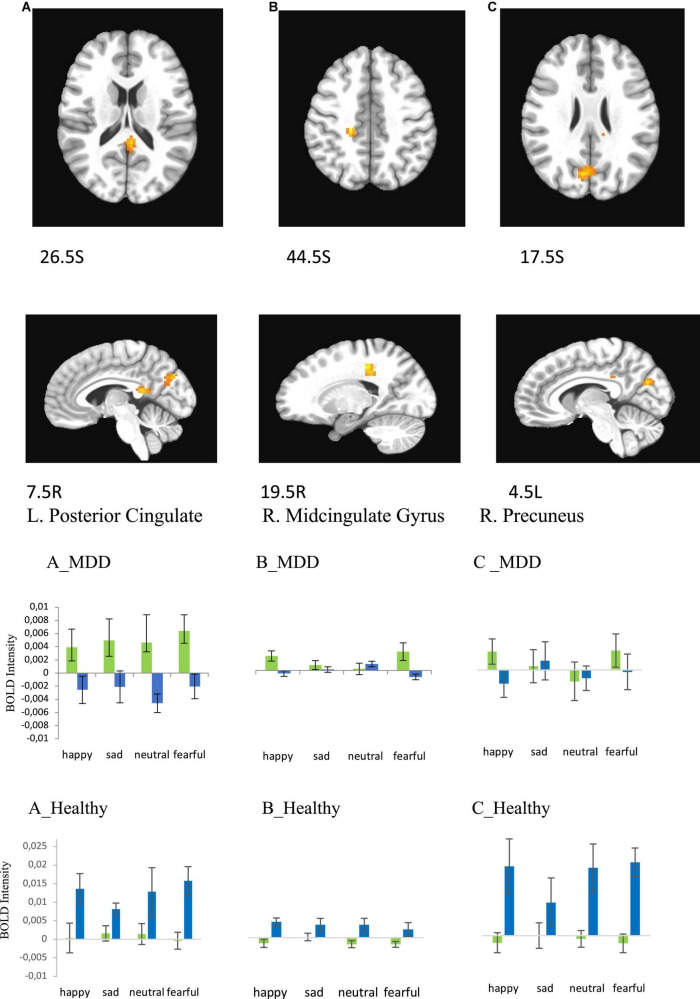
Brain regions that showed significant sex × group effect in MDD and Healthy participants for presentations of happy, sad, fearful, and neutral faces [females – green color, males – blue color; **(A)** left posterior cingulate, **(B)** right cingulate gyrus, **(C)** right precuneus (BA31)]. Plots for ROI that displayed a significant difference in BOLD intensity between females and males in MDD group [**(A–C)**, MDD group] and Healthy group [**(A–C)**, Healthy group] (*p* < 0.005).

Statistical analysis with 3dMVM in both the healthy and MDD groups revealed a significant sex × group interaction in three areas, the left posterior cingulate (*F* = 8.864, *p* = 0.005 for happy; *F* = 5.056, *p* = 0.030 for sad; *F* = 7.931, *p* = 0.007 for neutral, *F* = 22.927, *p* < 0.001 for fearful), right middle cingulate (*F* = 14.489, *p* < 0.001 for happy; *F* = 4.061, *p* = 0.050 for sad; *F* = 3.584, *p* = 0.065 for neutral, *F* = 7.139, *p* = 0.011 for fearful), and right precuneus (*F* = 18.745, *p* = 0.001 for happy; (*F* = 10.058, *p* = 0.003 for neutral; *F* = 17.854, *p* = 0.001 for fearful) areas (Figure 7 and Table 5). The activation levels in MDD women are higher than MDD men in left posterior cingulate (Figure 7A, MDD) but higher in healthy men than in healthy women (Figure 7A, Healthy). In contrast MDD women and men show similar activation levels in right midcingulate and right precuneus (Figures 7B, C, MDD) whereas healthy women show lower activation than healthy men (Figures 7B, C, Healthy).

**TABLE 5:** Regions with significant sex*group interaction effect in all participants.

Cluster #	Cluster size (# voxels)	Center of mass			Peak activation	Brodmann area
		**X**	**Y**	**Z**		
		**(Local peak activation)**				
1	67	−7.5	+73.5	+26.5	Right precuneus	
2	62	−19.5	+28.5	+44.5	Right cingulate gyrus	BA 31
3	38	+4.5	+43.5	+17.5	Left posterior cingulate	BA 30

#### IDS score and melancholic score correlations with BOLD signal intensity during emotional face processing

**FIGURE 8:**
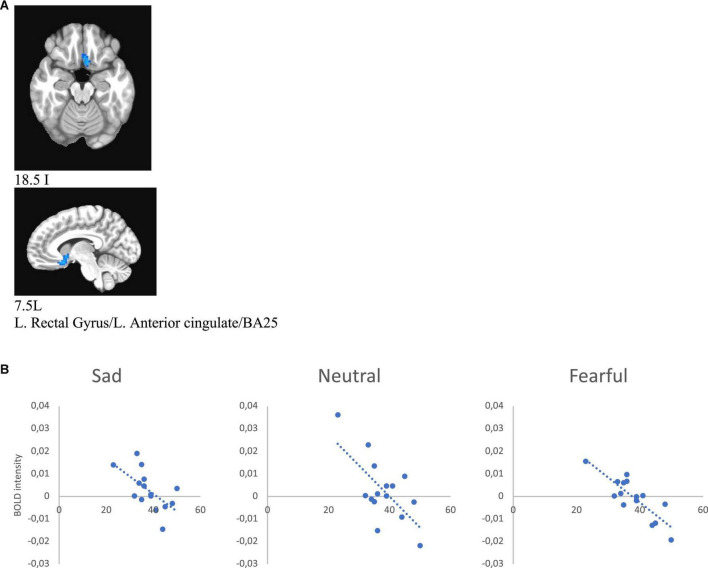
**(A)** Brain region that showed significant IDS score effect in female MDD participants [**(A)** subgenual cingulate/BA25). **(B)** Correlations of IDS score with BOLD intensity during sad (*r* = –0.616, *p* = 0.014), neutral (*r* = –0.668, *p* = 0.006), and fearful (*r* = –0.838, *p* < 0.001) faces perception.

Additionally, we ran 3dMVM analyses with IDS score and melancholic symptom score in the MDD group (*p* ≤ 0.005). A whole brain analysis was performed and significant clusters were extracted for an ROI analysis of correlates of these variables. This analysis showed a negative correlation between IDS score and BOLD intensity in the left rectal gyrus/left anterior gyrus/BA25/BA11 (for sad *r* = −0.616, *p* = 0.014, for neutral *r* = −0.668, *p* = 0.006, for fearful *r* = −0.838, *p* < 0.001) in female subjects, whereas there was no significant correlation between these variables in the male group (Figure 8 and Table 6).

**TABLE 6:** Regions with significant correlations between BOLD signal intensity during emotional face processing and IDS and melancholic scores in MDD group.

Cluster #	Cluster size (# voxels)	Center of mass			Peak activation	Brodmann area
		**X**	**Y**	**Z**		
		**(Local peak activation)**				
**BOLD signal and IDS score**						
1	50	+7.5	−13.5	−18.5	Left rectal gyrus Left medial frontal gyrus Left anterior cingulate	BA 11 BA25
**BOLD signal and melancholic score**						
1	87	+7.5	−4.5	+56.5	Left superior frontal gyrus	
2	46	−37.5	−19.5	+11.5	Right insula	BA 13
3	41	−40.5	+61.5	+23.5	Right middle temporal gyrus	BA 39

**FIGURE 9:**
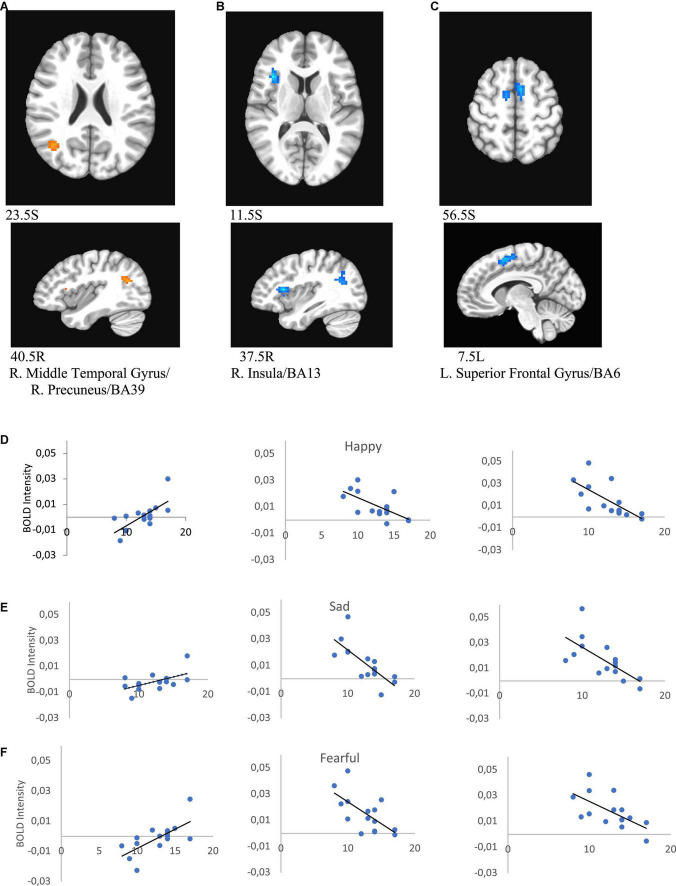
Brain regions that showed significant melancholic score effect in female MDD participants [**(A)** right middle temporal gyrus/right precuneus/BA39; **(B)** right insula/BA13; **(C)** left superior frontal gyrus/BA6). Correlations of melancholic scores with BOLD intensity during in panels **(A–C)** regions for happy **(D)**, sad **(E)**, and fearful faces **(F)** (*p* < 0.005).

Melancholic score was negatively correlated with activity in the left superior frontal gyrus/BA6 (for happy *r* = −0.690, p = 0.004; for sad *r* = −0.678, *p* = 0.005, for neutral *r* = −0.719, p = 0.003, for fearful *r* = −0.629, *p* = 0.012) and right insula/BA13 (for happy *r* = −0.648, p = 0.002; for sad *r* = −0.737, *p* = 0.002, for neutral *r* = −0.791, p < 0.000, for fearful *r* = −0.645, *p* = 0.009), and positively with activity in the right middle temporal gyrus/precuneus/BA39 (for happy *r* = 0.662, *p* = 0.007; for sad *r* = 0.641, *p* = 0.010, for neutral *r* = 0.600, *p* = 0.018, for fearful *r* = 0.673, *p* = 0.006) in female patients (Figure 9 and Table 6).

## Discussion

The present study investigated differences in brain activation in response to implicit perception of facial emotions in healthy participants and patients with MDD who met the criteria for the melancholic features specifier according to the DSM-IV. Mixed support for hypotheses was obtained. Confirming our first hypothesis, we found decreased prefrontal cortical activation in multiple areas for MDD patients, but there was no evidence for greater limbic or paralimbic activation in patients. Second, we obtained limited support for mood-congruence in group differences in response to faces of differing emotional valence. There was no evidence for mood-congruence in response in frontal areas, but patients showed stronger response to fearful faces than to happy faces in middle temporal gyrus. Third, we did not find that female patients showed generally stronger indications of depression than men in fMRI data, but there was limited support for the hypothesized greater frontal deactivation in women in the left middle frontal gyrus. Fourth, correlational analyses identified several regions in which response strength was related to the severity of illness as assessed by rating scales of depressive symptoms. We also obtained several unanticipated findings including sensitivity of areas of cerebellum (culmen and declive) to emotion and sex differences in MDD patients. Overall, data suggest that frontal hypoactivation is the most salient feature of melancholic depression in both men and women, with additional features of depression appearing more limited in scope.

The finding of cortical hypoactivation in patients experiencing a major depressive episode is compatible with reports from previous studies (Mayberg, 1997; Keedwell et al., 2010; Stuhrmann et al., 2011; Zhong et al., 2011; Zhang et al., 2021). Specifically, we found decreased brain activity in response to facial emotions, irrespective of emotional valence, in the left and right superior frontal gyrus, and right middle frontal gyrus (BA8) in patients with depression compared to healthy participants. The left superior frontal and middle frontal gyri are also associated with cognitive control of emotional state, especially frontal regulation of the amygdala (Frank et al., 2014; Wang et al., 2019). Furthermore, decreased activation in the right supramarginal gyrus, which has been implicated in emotion-regulation and cognitive reappraisal of negative emotive stimuli (Ochsner and Gross, 2014; Loeffler et al., 2019), is consistent with dysregulation in depressed individuals who attend to negative rather than positive stimuli, interpret ambiguous stimuli negatively, and selectively recall negative memories (Leppänen, 2006; LeMoult and Gotlib, 2019).

Our behavioral and imaging data partly supported a mood-congruent emotional bias in MDD patients exhibited by increased sensitivity to negative stimuli (Sheline et al., 2001; Stuhrmann et al., 2011). We observed no group by emotion interaction. However, significantly decreased activation to happy faces and increased activation to fearful faces was observed in the right middle temporal gyrus in depressed participants only. It is known that the middle temporal gyrus involved in face and emotion recognition (Pourtois et al., 2005; Borghesani et al., 2019). At the same time behavioral data showed slower reaction time to fearful faces in the depressed group as well. This localized effect contrasts with evidence for more extensive mood-congruence of response to negative facial and other stimuli in depressed patients documented in several major reviews and meta-analyses (Groenewold et al., 2013; Li and Wang, 2021; Pizzagalli and Roberts, 2022). The present study showed neither the enhanced limbic and paralimbic response to negative stimuli noted by these authors, nor hypoactivation of frontal areas including DLPFC. Mood-congruence of brain response is less pronounced in melancholic depression than in other subtypes. The implicit nature of the emotion manipulation (Gur et al., 2002b) may also play a role. Limbic system response appears to be stronger for explicit than for implicit emotional stimuli (Habel et al., 2007), such that the emotional content of the current task may not have been sufficiently salient to activate mood-congruent responses in structures such as amygdala and ventral ACC. Similarly, the implicit task may have limited differences in emotion-regulation following negative stimulus presentation associated with depression, especially voluntary emotion control strategies supported by DLPFC (Rive et al., 2013). The present data suggest that, even with implicit stimuli, melancholic depression is associated with frontal hypoactivation, but use of explicit emotion tasks may be needed to investigate the spectrum of mood-congruence effects linked to MDD.

With regard to sex differences, female participants with MDD exhibited increased BOLD intensity compared to male MDD participants in posterior cingulate cortex consistent with observed sex differences in healthy participants (Wager et al., 2003). The negative correlation between melancholic scores and BOLD intensity in the right insula and left superior frontal gyrus to happy, sad, and fearful faces in women with MDD may indicate sex specificity in emotion processing dysregulation in depression. Emotional face processing has been used to examine reaction time and brain activity of the emotion regulation system to predict symptom improvements after different treatments in depressed patients (Roseman et al., 2018; Stroud et al., 2018; Gorka et al., 2019; Fisher et al., 2022). In fact, diminished insula activation is a reported characteristic of MDD (Grimm et al., 2009). The effects of depression on cingulate regions and insula may primarily be linked to emotion appraisal rather than emotion-regulation (Etkin et al., 2011; Groenewold et al., 2013; Li and Wang, 2021). Therefore, some emotional processing differences between women and men with depression may be closely associated with differences in emotion appraisal processes. As for mood-congruence, we might anticipate additional sex differences in brain response if an explicit rather than an implicit task was used.

Of note, we found a correlation between depression severity and BOLD intensity in the subgenual gyrus/anterior cingulate area (BA25) in women. Previous studies have shown that the degree of change in activity in this region during antidepressant treatment is related to the level of symptom reduction (Hamani et al., 2011). BA25 has been studied as an implantation site for deep brain stimulation, an investigational treatment for MDD (Hamani et al., 2011; Hall et al., 2014). Furthermore, evidence supports a link between BA25 and hypothalamic-pituitary-adrenal (HPA) axis activity (Sudheimer et al., 2013). Interestingly, there are reports that HPA axis dysregulation in depression differs between depressive subtypes: patients who exhibit melancholic symptoms are characterized by hypercortisolism, whereas the presence of atypical symptoms has been linked to decreased (HPA) axis activity (Geracioti et al., 1997; Lamers et al., 2013). Data obtained using single photon emission computed tomography (SPECT) to assess functional brain activity in depression suggest there is decreased right frontal perfusion in patients with melancholic compared with atypical depressive symptoms (Fountoulakis et al., 2004). Our finding of a negative correlation between melancholic symptoms and left superior frontal gyrus (BA6) and right insula (BA13) activity support a possible link between the melancholic subtype and decreased cortical activity.

Several limitations of the study should be emphasized: the results were obtained from a homogenous group of MDD patients and deserve to be repeated in other subsets of depressed patients such as depression with atypical features, which is characterized by reverse vegetative symptoms and is over-represented in women (Shuch et al., 2014) in order to determine the specificity of our current findings. The majority of previous studies have focused on tasks requiring explicit recognition of emotion. The present study is novel in investigating implicit processing of emotion during a gender discrimination task, but more research is needed to determine similarities and differences between implicit and explicit emotional processing in depressed patients. Also, our small sample size limits the generalizability of our findings related to neural dysregulation of emotional processing-related deficits in depression.

## Conclusion

Brain activation differences during implicit emotional processing exist in patients with depression with melancholic features and may contribute to phenomenological presentations in MDD. The present findings showed influences of MDD with melancholic features on brain systems associated with both appraisal and emotion-regulation. Sex differences were most prevalent in posterior cingulate cortex, a structure linked to appraisal of emotive stimuli The current study results may help to clarify how specific regions of the brain provide markers of depression symptoms associated with implicit emotion processing, supporting therapies informed by understanding of abnormalities in brain functioning.

## Data availability statement

The datasets presented in this article are not readily available because restrictions apply to the datasets. The datasets presented in this article are not readily available because our IRB protocol prohibits the public release of individual datasets. Requests to access the datasets should be directed to EN at nelsoneb@ucmail.uc.edu.

## Ethics statement

The studies involving human participants were reviewed and approved by the Institutional Review Board (IRB) of the University of Cincinnati. The patients/participants provided their written informed consent to participate in this study.

## Author contributions

AK was involved in the behavioral and fMRI data analysis, and writing the manuscript. JE and EN contributed to the designing and conducting the research protocol, providing funding for the research, conducting the study, acquiring and processing the data, and revising the manuscript. GM was involved in the literature review and revision of the manuscript. All authors contributed to the article and approved the submitted version.

## Footnotes

1
www.pstnet.com
